# Design of FRET Probes for SNP RS1006737, Related to Mood Disorder

**DOI:** 10.2174/1745017901814010053

**Published:** 2018-02-28

**Authors:** Germano Orrù, Mauro Giovanni Carta, Alessia Bramanti

**Affiliations:** 1 Department of Surgical Sciences, Molecular Biology Service (MBS), University of Cagliari, Cagliari, Italy; 2 National Research Council of Italy, ISPA, Sassari, Italy; 3 Department of Medical Sciences and Public Health, University of Cagliari, Cagliari, Italy; 4 Istituto di Scienze Applicate e Sistemi Intelligenti, ISASI, Messina, Italy

**Keywords:** *Mood disorders*, *Genome-wide association studies (GWAS)*, *CACNA1C gene*, *RS1006737*, *FRET probes*, *Real time PCR*

## Abstract

**Background:**

Several studies have shown that the Single Nucleotide Polymorphism (SNP) in the CACAN1C gene, rs1006737, is related to different mood disorder illnesses, such as bipolar disorder and schizophrenia. Current day molecular procedures for allele detection of this gene can be very expensive and time consuming. Hence, a sensitive and specific molecular procedure for detecting these mutations in a large number of subjects is desirable, especially for research groups who have no complex laboratory equipment.

**Objective:**

The possibility of using a Fluorescence Resonance Energy Transfer (FRET) probe was evaluated by means of bioinformatic tools, designed for forecasting the molecular behavior of DNA probes used in the research field or for laboratory analysis methods.

**Method:**

In this study we used the DINAMelt Web Server to predict the *T*ms of FRET oligo in the presence of the A and/or G allele in rs1006737. The PCR primers were designed by using oligo 4 and oligo 6 primer analysis software,

**Results:**

The molecular probe described in this study detected a *T*m difference of 5-6°C between alleles A and G in rs1006737, which also showed good discrimination for a heterozygous profile for this genomic region.

**Conclusion:**

Although *in silico* studies represent a relatively new avenue of inquiry, they have now started to be used to predict how a molecular probe interacts with its biological target, reducing the time and costs of molecular test tuning. The results of this study seem promising for further laboratory tests on allele detection in rs1006737 region.

## 1 INTRODUCTION

Although the laboratory molecular approach in Mood Disorders (MD) has currently no clear diagnostic value, the need for its use in an “omics comprehension” of this illness has become more and more obvious. In fact, MD and also bipolar disorders and sub-threshold mania conditions are regulated in the patient by multifactorial, genetic and epigenetic factors [1-3], in which transcriptomics, proteomics, metabolomics and environmental conditions work closely together at disease onset and during its progression [4]. This is the context in which it is hoped that further integration of these techniques will yield a more comprehensive understanding of mood disease etiology and its biological pathways.

In recent years, the use of Genome-Wide Association Studies (GWAS) has provided an extensive investigation of genomes in healthy and sick patients and these results could thus prove promising in the future for an extensive comprehension of BD-associated genetic features [5, 6]. Research carried out on a very large human population has highlighted numerous genetic variants/mutations in both coding and in non-coding gene regions. Some of these appear promising for suggesting a significant association between MD and DNA changes [7, 8]. This work only focuses on a Single Nucleotide Polymorphism (SNP) located in the CACNA1C gene (rs1006737), which is one of the most studied and replicated mutations associated to mood disorders. We are aware that this is an arguable choice, but a study of all the mutations in the candidate genes reported so far is beyond the scope of this article. In fact, the aim of this work is the valuation *in silico* of a molecular probe able to detect in a short time the allelic variations previously described in rs1006737 as regards the risk of MD for this genic region.

### 1.1 CACNA1C Function and Gene Structure

This gene, located in chromosome 12, encodes an alpha-1 subunit of a voltage-dependent calcium channel that mediates the influx of calcium ions into the cell, upon membrane polarization. It consists of 24 segments positioned in a cell transmembrane space, able to form a pore for calcium transfer. This molecular structure is responsible for calcium influx in response to membrane depolarization, thereby regulating different intracellular processes such as neurotransmission and gene expression in many different cells and neurons. This activity results as being essential for coupling electrical signals on the cell surface to physiological events in the cells [9, 10]. The complete calcium channel is structured as a complex of alpha-1, alpha-2/delta, beta, and gamma subunits in a 1:1:1:1 ratio. As has been previously described in other publications, an alternative splicing also exists that results in numerous transcript variants which encode different proteins, but no such products result for functional ion channel subunits [11]. In the gene sequence corresponding to GenBank accession n. NG_008801, CACAN1C takes up a region of 728,273 bp and 51 exons Fig. (**1**). In variant 4, the mRNA is about 13500 bp long (Gen Bank accession NM_001129830), Fig. (**1**).

### 1.2 Risk Allele RS1006737

To date, this variant represents the most cited and studied genetic risk for bipolar disorders, but it also confers a risk of recurrent major depression and schizophrenia [12-18]. As shown in Fig. (**1**), the SNP is located in the intronic region of CACNA1C, Fig. (**1**). The A allele is thought to be a risk factor and various studies have shown its odds ratio as ranging between 1.18-1.07 [19-21]. Some researchers have hypothesized that this variant is able to influence gene expression, and others have demonstrated an effective variation of CACNA1C mRNA in post-mortem brain studies. The frequency of this allele appears to be strictly correlated to the ethnic origin of the analyzed population, for example, about 54% in African descent while 5% in the Asian population. Moreover, different discrepancies exist as regards the interpretation of molecular data in the clinical findings, which is also due to the result of these allelic differences in different ethnical groups and an admixture of these in some countries, *e.g*. in the USA population. Thus, some researchers have found no effects, while others have reported a significant reduction in cognitive function [16, 22, 23], but only in individuals who carried the homozygous A allele. Due to the lack of clinical specificity, such controversies require further and extensive studies, and in particular, this nucleotide profile could be detected by faster, less expensive molecular procedures than those used in current molecular analysis. In light of these facts, we thus performed an *in silico* study to investigate the suitability of Fluorescence Resonance Energy Transfer (FRET) technology for use in DNA probes to detect G-A variants in rs1006737 in human samples.

### 1.3 Usefulness of FRET Probes for Allelic Profile Analysis

FRET phenomena are observable when two dye molecules are in close proximity (about 1-5 nucleotides apart), and in this condition, due to an energy transfer from one molecule (donor) to the other (acceptor), the latter may show instability with photon emission [24]. FRET technology has been used to investigate a variety of biological phenomena that produce changes in molecular proximity, such as nucleotide variations in a DNA fragment [25-27]. FRET dyes could be applicable in DNA probes structured as shown in Fig. (**2**). Two fluorophores are bound in 2 different DNA fragments that are complementary to the DNA sequence under study, the first one in the 3' position of the fragment and the second one in the 5' position of the other adjacent oligonucleotide. These dyes were chosen so that the emission spectrum of one significantly overlaps with the excitation spectrum of the other. During FRET, the donor fluorophore, excited by a light source, transfers its energy to an acceptor fluorophore when positioned in the direct vicinity of the former, thus the probe system consists of two oligonucleotides labeled with two different fluorescent dyes. During a PCR reaction, the oligonucleotides hybridize to complementary regions of the DNA target and the fluorophores, which are coupled to the oligonucleotides, are in close proximity, Fig. (**2**). The donor fluorophore (F1) is excited by an external light source from the PCR thermocycler and then passes part of its energy to the adjacent acceptor fluorophore (F2). The latter dye emits light at a different wavelength which can then be detected and measured by a PCR machine. This technique can be applied to genotype variants in rs1006737 by using melting curve procedures during the PCR reaction and the subsequent melting temperature by the melting peak interpretation, which is now evaluable by means of online tools such as the DINAMelt program [28].

## 2 METHODOLOGY

We analyzed the CACNA1C 480 bp fragment corresponding to GenBank accession n. NG_008801 (from nucleotide 270121 to 27600) indicating the normal allele (G), Fig. (**1**). This DNA sequence was analyzed by using the “mfold Web Server” tools available on line from the RNA Institute (University at Albany, State University of New York), http://www.albany.edu/rna/. The folding of the sequence was studied to avoid the presence of high energy DNA loops able to interfere with FRET probe positioning during the PCR reaction [29]. Subsequently, DINAMelt was used to design the probe and to study the prediction of the melting temperatures (*T*ms) in the presence of the different rs1006737 alleles G and A.

### 2.1 Tm Prediction and Probe Structure

The range of *T*m events depends on different conditions: (i) probe length, (ii) GC% content, (iii) the molar concentration of monovalent cations, (iv) steric hindrance of the fluorophore bound to the oligo [26]. The DINAMelt tool was used with chemical and physical parameters for a PCR reaction ([Na]= 50 mM and [Mg] = 2 mM). The temperature range was set from 50°C to 100°C. The theoretical concentration of the probes was 20 µM [27, 28]. The *T*m values compared to the length of the acceptor probe for both alleles A and G has been evaluated, they are shown in Fig. (**3**).

### 2.2 PCR Design

We designed a real time PCR reaction by using the Oligo 6 and Oligo 4 programs (Med.Probe, Oslo, Norway) as described in other publications [30-47].

## 3 RESULTS AND DISCUSSION

Some authors have reported real time PCR procedures, by using TaqMan probes, to detect rs1006737 SNP in the CACNA1C gene [48]. This molecular probe consists of an oligonucleotide with a fluorescent reporter at one end, and a fluorescence quencher molecule at the opposite end of the probe. The proximity of these fluorophores prevents the emission of fluorescence, but the hydrolyzation of the probe, caused by the 5' to 3' exonuclease activity of the Taq polymerase during the PCR reaction, releases the reporter and thus allows fluorescence emission. Although this procedure is easy to use and has been indicated by many authors for genotyping, other molecular works carried out with reconstruction experiments, have demonstrated its limited use in terms of accurate discrimination in SNP detection, especially as far as heterozygotes are concerned. In fact, this condition has proved to be a problematic point in PCR based genotyping, in this case with the AG allele. An alternative method in rs1006737 genotyping with greater specificity/selectivity could be to use a molecular procedure based on FRET probes. In comparison with traditional or recent molecular methods, such as RFLP or DNA sequencing procedures, this approach resulted as being faster, less expensive, simpler and accurate [49]. In FRET technologies classic Dye chemistry are composed from fluorescein isothiocyanate (F1) at 3′ end of upstream probe, while the downstream probe (acceptor) is labeled with the fluorophore Red 640 (F2), Fig. (**2**), and (**3**). A single G-A variation can be detected in rs1006737 because the nucleotide change in the DNA target destabilize binding of the acceptor probe and, hence, cause a characteristic decrease in the melting temperature (*T*m). In the PCR apparatus this result is displayed by a melting peak shift, by monitoring the F2 fluorescence during sampling heating, Fig. (**4**). Our work, which was exclusively performed *in silico,* has indicated a good possibility of allelic discrimination for this gene region by melting temperatures evaluation.

Fig. (**3**) summarizes results from studies that have sought to detect the differences between melting temperature *T*ms in the presence of the G or A allele in SNP rs1006737 as compared to the respective nucleotide length of the acceptor probe. In accordance with the data obtained by the DINAMelt program, the optimal length was assessed at 16 nucleotides when the Δ*T*m resulted as being 5°C, a value which could be sufficient to discriminate between the two allelic profiles. In particular this approach it may be able detect the allele A, which has been indicated as a risk in bipolar disorders and Schizophrenia [50]. In our previous works we have assessed that the maximum difference in *T*m between: (i) theoretical and (ii) real values, using real time PCR procedures by using different FRET probes, corresponds to 0.5°C max [25, 26], in these publications, we also noted that a good rate of sensitivity/specificity of this method was observed with two rounds of DNA amplification [51]. In fact Table (**1**) indicates the theoretical PCR oligos designed for a nested PCR suitable for this FRET SNP detection. Fig. (**4**) shows the melting peaks obtained with DINAMelt, simulating a PCR platform such as a LightCycler (Roche). The mutation analysis by Next Generation Sequencing or by real time PCR with TaqMan probes are a widely used method to differentiate A and G allele in SNP rs1006737. However in comparison with proposed FRET method the first one is a relatively time-consuming and many expensive procedure (about 100-200 fold higher) and it require a high minimum number of samples for to be cost efficiency [52]. TaqMan could be unable to distinguish the presence of both G-A alleles with errors in specificity and selectivity (heterozygous profile) [51].

Thus, this procedure could be achievable within a molecular procedure able to detect the nucleotide variation in rs1006737 by means of a fast-real time PCR laboratory protocol.

## CONCLUSION

Although the implications of our results are theoretical, we are able to assert that FRET probes could be employed in laboratory tests for the detection of rs1006737 alleles, for example, by using patient’s saliva DNA. In further studies, it could be interesting to evaluate whether other SNPs in CACANA1C, or in other MD-related genes, are detectable by different FRET oligos. This could be a molecular model for a non-expensive routine procedure for multiple MD-related SNPs, which have already been described in clinical studies with more complex molecular analyses, such as those using Next Generation Sequencing (NGS).

## FOOTNOTES/WEB LINKS

- GeneCards: http://www.genecards.org/cgi-bin/carddisp.pl?gene=CACNA1C
- SNpedia: https://www.snpedia.com/index.php/Rs1006737
- Entrez: https://www.ncbi.nlm.nih.gov/SNP/snp_ref.cgi?rs=1006737
- CACNA1C: https://www.ncbi.nlm.nih.gov/nuccore/NG_008801.2
- DINAMelt: http://unafold.rna.albany.edu/?q=DINAMelt

## Figures and Tables

**Fig (1):**
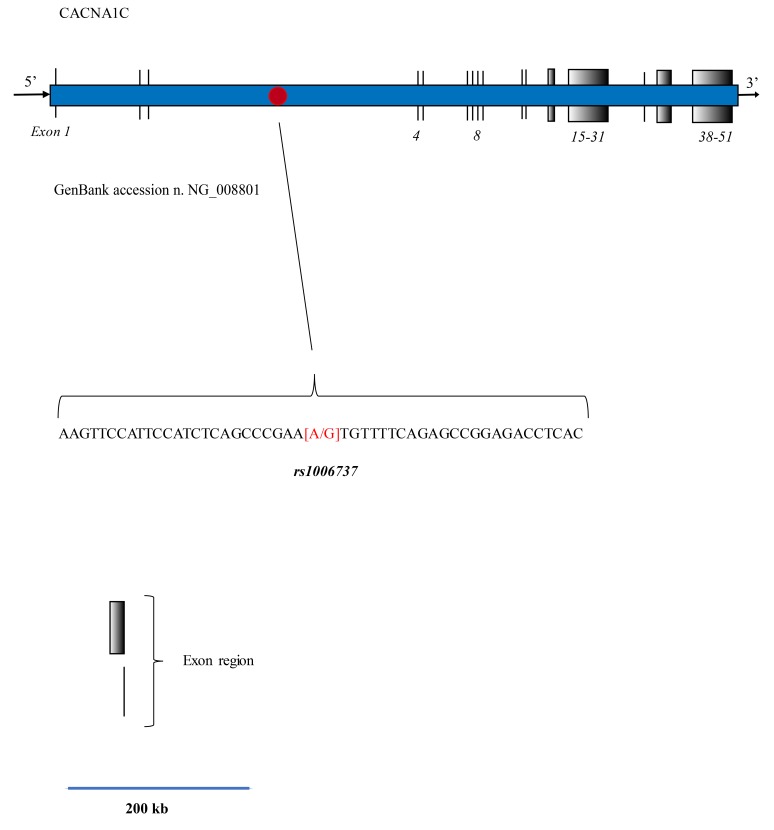
Position of rs 1006737 in the CACAN1C gene associated to mood disorders, following recent genome-wide association studies (GWAS)[53].

**Fig (2):**
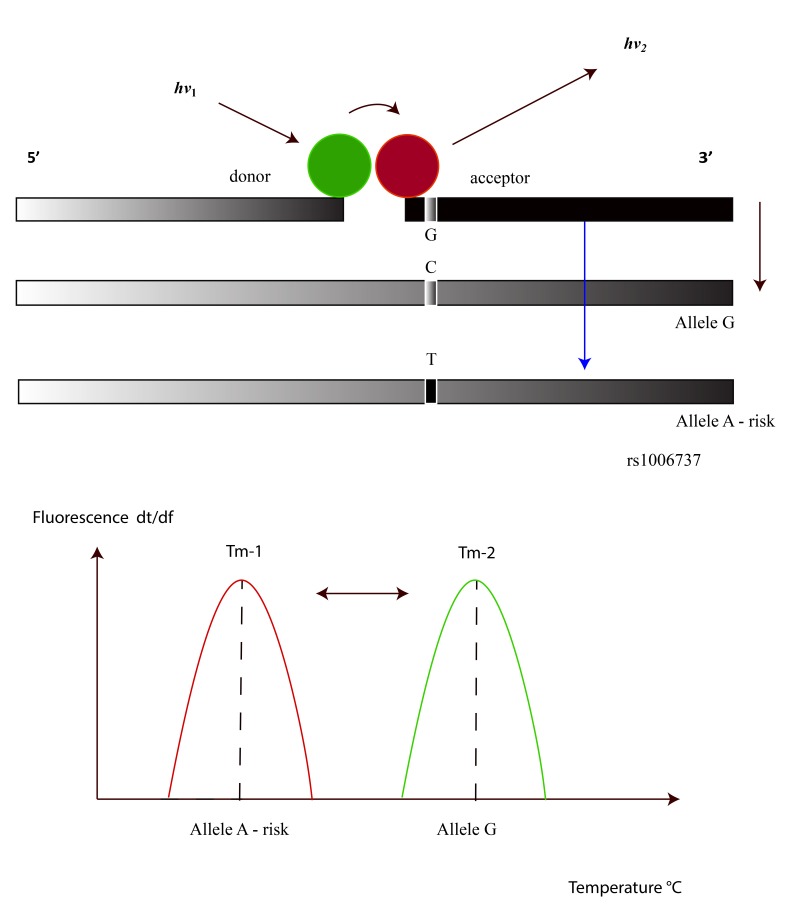
Schematic representation of FRET probe structure and the possible melting peak array with rs1006737 alleles.

**Fig (3):**
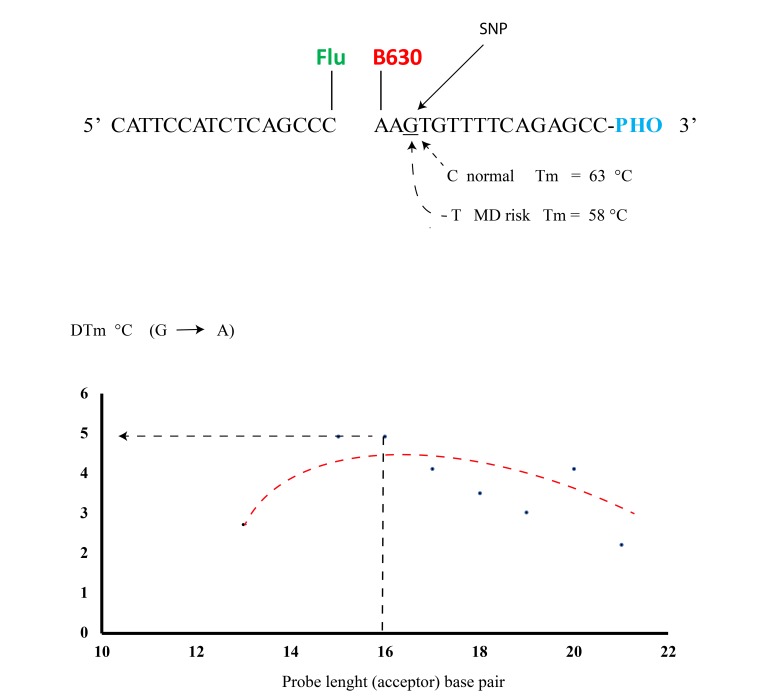
Sequence and structure of the FRET system valuated in this study. The typical ROCHE Dye, Fluorescein and LC 640, are indicated in the figure, PHO - phosphate. The graph represents the tendency curve indicating the donor probe length and *T*m differences between the A and G variants in rs1006737.

**Fig (4):**
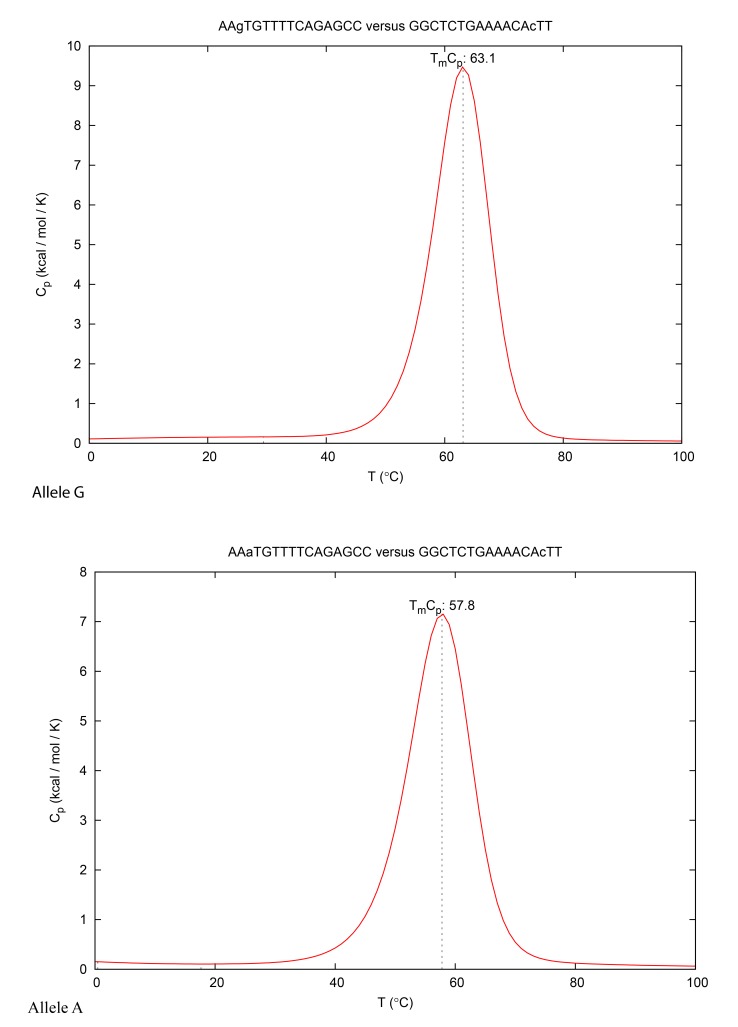
*In silico* forecast analysis for the allelic discrimination between A and G alleles for SNP rs1006737 by using FRET probes in real time PCR after melting curve real time PCR protocol.

**Table 1:** Oligonucleotides designed for a nested real time PCR for rs1006737.

**Oligo Name**	**Sequence 5'-3'**	**Length (nt)**	**Position** NG_008801
OG644F	GGCTTCAGAGTCCACTTGGC	20	270271
OG645R	TGGGCACATTCAAACCTGAA	20	270410
OG646F	CAAAGTCTTGCTATCAATTACATA	24.	270296
OG647R	CTGAGAGACACTGTGAGGTC	20	270380
OG648_Fam_	CATTCCATCTCAGCCC^Fam^	16	270325
OG649_R640_	^Red640^AAGTGTTTTCAGAGCC^PHO^	16	270342

**Note:** 644-645 = external PCR, amplicon length = 140 bp

646-647 = internal PCR, amplicon length = 85 bp

Theoretical annealing temp. = 50°C for both reactions.
